# The erosion of ambiguity tolerance and sustainment of perfectionism in undergraduate medical training: results from multiple samplings of a single cohort

**DOI:** 10.1186/s12909-020-02345-5

**Published:** 2020-11-10

**Authors:** Silvio Ndoja, Saad Chahine, Donald H. Saklofske, Brent Lanting

**Affiliations:** 1 Schulich School of Medicine & Dentistry, University of Western Ontario, 1151 Richmond St, London, ON N6A 5C1 Canada; 2 Faculty of Education, Queen’s University, Duncan McArthur Hall, 511 Union St W, Kingston, ON K7M 5R7 Canada; 3 Department of Psychology, University of Western Ontario, 1151 Richmond St, London, ON N6A 5C1 Canada; 4 London Health Sciences, 339 Windermere Rd, London, ON N6A 5A5 Canada

**Keywords:** Tolerance of ambiguity, Perfectionism, Clerkship, Medical students

## Abstract

**Background:**

Medicine is a field that is simultaneously factual and ambiguous. Medical students have their first exposure to full time clinical practice during clerkship. While studies have examined medical trainees’ tolerance of ambiguity (TOA), the extent to which TOA is affected by clinical experiences and its association with perfectionism is unknown. The aim of this study was to evaluate the effect of clerkship experience on TOA and perfectionism in medical students.

**Methods:**

This was a multiple sampling, single cohort study of students in their first year of clinical clerkship which is comprised of 6 core rotations. Consenting students completed an online anonymous survey assessing their tolerance of ambiguity (TOA) and perfectionism in their first (pre) and last (post) 12 weeks of their clinical clerkship year. Tolerance of Ambiguity in Medical Students and Doctors (TAMSAD) and The Big Three perfectionism scale-short form (BTPS-SF) were used to assess TOA and perfectionism respectively. Pre-Post mean comparisons of TOA and perfectionism were assessed via t-tests.

**Results:**

From a cohort of 174 clinical clerkship students, 51 students responded to pre-survey, 62 responded to post-survey. Clerkship was associated with a significant decrease in TOA (*p* < 0.00) with mean pre-TOA scores of 59.57 and post TOA of 43.8. Perfectionism scores were not significantly different over time (*p* > 0.05). There was a moderate inverse correlation between TOA and perfectionism before clerkship (r = 0.32) that increased slightly after clerkship (r = 0.39). Those preferring primary care specialties had significantly lower rigid and total perfectionism scores in pre-clerkship than those choosing other specialties, but this difference was not found post-clerkship.

**Conclusion:**

Exposure to clerkship decreased TOA while perfectionism remained stable in medical students. These results were not expected as exposure has been previously shown to increase TOA. The frequency of rotation changes maintaining a cycle of anxiety may be an underlying factor accounting for these results. Overall these results require further investigation to better characterize the role of clinical exposure on TOA.

## Background

Becoming a practicing physician requires the completion of an intensive and demanding residency program. In the final years of medical school, students must choose which of the 30 direct entry specialties they wish to specialize in as a career. Many factors can influence a student’s choice in career paths including lifestyle, location, and “fit” [1, 2]. The final years of medical school, also known as the clerkship years, are typically comprised of medical students getting first-hand experience in various medical fields. This serves a two-fold purpose: to create a comprehensive foundation of medical knowledge and to expose them to possible specialization career choices.

Each specialty can vary in the degree of patient information available to practitioners. Patients can present with vague, non-specific symptoms, such as diffuse abdominal pain, or exhibit clear presentations such as a fracture of the humerus due to a fall—or anywhere in between that spectrum. Different presentations are associated with different degrees of ambiguity in diagnoss, treatments, and prognosis. As such, different medical specializations and contexts may potentially be better suited to certain personality traits. Both tolerance of ambiguity and perfectionism are personality traits that vary across all persons, but not frequently researched in the context of medical education and thus are the focus of this study [3–5].

Tolerance of ambiguity (TOA) refers to how we perceive, respond to, and tolerate information that may lack credibility, and is uncertain [6–9]. While interest in TOA in the medical field can be traced back to the early 1990s, studies have often led to conflicting results, with some showing a larger, but non-statistically significant, TOA in 3rd-year medical students (first year of clerkship experience in North American schools) [6] and others showing no difference [10]. However, a recent review of 11 studies (Hancock and Mattick, 2020) concluded there was an association between a lower level of tolerance of ambiguity and lower psychological well-being amongst medical students and practicing doctors [3]. Residents have been reported to have higher TOA compared to medical students [11]. When examining baseline data of 13,867 matriculating first-year medical students in the United States in 2013, higher TOA was seen in men and older individuals [12]. Interestingly, there was a statistically significant relation between TOA and declared specialty of interest; those students pursuing specializations in Dermatology, Physical medicine, and rehabilitation, and otolaryngology have the lowest mean TOA scores in contrast to those selecting Psychiatry, Radiation Oncology, Emergency medicine, and Neurosurgery the highest TOA [13]. However, this was an incidental finding and no further analyses were done. Other studies have shown that surgeons have a lower TOA than other physicians [14]. However, we are not aware of research investigating how TOA changes with clinical exposure.

Perfectionism is a personality trait of interest in the medical field as it has been implicated in anxiety [15, 16], depression [17], and burnout [18–20]. While perfectionism may appear to have an intuitive definition, it is emerging to be a multi-dimensional construct. Although there are several models describing the composition of perfectionism, recent research has identified three measurable dimensions: rigid perfectionism, self-critical perfectionism, and narcissistic perfectionism [21]. Rigid perfectionism is defined as requiring “flawless performance from the self” [22], self-critical perfectionism is defined as negative responses to flawed performances [23], and narcissistic perfectionism refers to expecting perfectionism from others in a grandiose, hypercritical, and entitled way [24]. Medical students have been shown to have higher perfectionism scores than arts students, with maladaptive perfectionism being predictive of depression and academic distress [25], a result that has been replicated in other studies [18, 26, 27].

More recently, Leung et al. (2019) described a personality profile of medical students linking TOA and high levels of maladaptive perfectionism, which in turn may underlie vulnerability to stress and ineffective coping [4]. However, we are not aware of any studies investing how perfectionism may change with clinical training and experience and its relationship with TOA.

The aim of this study was guided by three research questions: 1) To what extent does relationship exist between TOA and perfectionism for medical students in their first year of clerkship? 2) How does clerkship modify these factors and/or their relationship? 3) Are perfectionism and TOA related to a student’s specialty choice?

## Method

### Participants and setting

All medical students in their clerkship year at our local institution during the period August 27th, 2018 to August 9th, 2019 were invited to participate in an anonymous survey assessing these personality traits. Clerkship at our instution occurs during the 3rd year of medical school and is comprised of 6 broad specialties with opportunities of subspecialty exposures where applicable [28]. The Qualtrics™ online survey was comprised of questions requesting demographics information (gender and age), the TAMSAD and BTPS-SF questionaires, and a list of all medical specialties where students were asked to rank their preferred top 5 specialties. The survey was distributed to eligible applicants through the school’s undergraduate medical education (UME) office, again with the participant’s being anonymous to comply with UME policies. This was a repeated single cohort pre and post-study. The survey was distributed at 2 times points: the start of the first rotation (pre-clerkship) and again at the start of the last rotation (post-clerkship). Participants were required to complete all questions in the survey. At each distribution, students who had not completed the survey received a 2-week reminder. Students were offered token of appreciation of $5 electronic gift card for each survey they completed for a total of $10.

### Measures

The Tolerance of Ambiguity in Medical Students and Doctors (TAMSAD), developed by Hancock, et al. (2015), is a 29 item Likert scale that specifically assess ambiguity in clinical situations [11]. This scale has good internal reliability and its use of clinical scenarios makes it an appealing tool for this study.

The 16 item Big Three Perfectionism Scale–Short Form (BTPS-SF; Feher, Smith, and Saklofske, 2019) assesses three broad factors: rigid perfectionism, self-critical perfectionism, and narcissistic perfectionism [29]. The short form has demonstrated good internal consistency and retest reliability. Confirmatory factor analysis supports the three factor structure of the well-validated 45 item scale [24]. The short form was chosen for its ease of use and sound psychometric properties.

### Data analysis

To ensure the anonymity of participants, it was not possible to match the participants’ responses on the two administrations. Thus, data analyses were limited to descriptive statistics and group comparisons rather than comparisons of medical students across time. As well, the anonymous nature of data collection did not allow us to determine number of participants lost to follow up or new participants who later opted into the study. Data were analyzed with SPSS and pre- and post-test groups were compared using t-tests [30].

The relationship between TOA and perfectionism with specialty choice was assessed by grouping the top 3 specialties. This was done because of the lower than anticipated response rate, but also because family medicine, internal, and emergency medicine can be classified as primary care specialties. This combined group was compared to other remaining specialties. The pre and post surveys were analyzed separately.

## Results

Of the total cohort of 174 students, 51 completed the pre- and 62 completed post-clerkship surveys that were retained for analysis. The gender split was somewhat comparable at time 1 (m = 24, f = 27) and time 2 (m = 28, f = 34). The difference in participant ages at time 1 (m = 25.31 years, s.d. = 2.18) and time 2 (m = 26.00 years, s.d. = 2.02) was about 2/3rd of a year.

Overall specialty preferences remained stable in the pre and post surveys (Table 1). Family medicine, Internal medicine, Emergency medicine, Anesthesiology, and Pediatrics remained the top 5 most popular specialties from the start and end of clerkship. Family medicine remained the top with 15/51 ranking it first in the pre-survey and 15/63 in the post-survey.

**Table 1:** Top 5 most popular specialties pre-clerkship where *n* = 51, and post-clerkship where *n* = 63 (post-clerkship response in brackets)

Specialty	Rank 1	Rank 2	Rank 3	Rank 4	Rank 5
Family Medicine	15 (15)	13 (15)	4 (3)	6 (1)	5 (3)
Internal Medicine	8 (5)	4 (5)	9 (7)	6 (5)	5 (5)
Emergency Medicine	5 (3)	7 (12)	7 (3)	8 (9)	4 (2)
Anesthesiology	2 (1)	3 (2)	5 (5)	5 (5)	3 (2)
Pediatrics	4 (6)	6 (0)	0 (5)	3 (3)	2 (3)

There was a statistically significant inverse correlation between perfectionism and TOA at time 1(− 0.32, *p* < 0.05) (Table 2). This result was maintained in the post-survey (− 39, *p* < 0.01). Of interest was that narcissitic and rigid perfectionism were significantly correlated with TOA only at the post survey data collection, whereas self-critical perfectionism showed a non-significant correlation with ambiguity tolerance at both times.

**Table 2:** Relationship between perfectionism and TOA pre-clerkship and post-clerkship (post-clerkship relationship in brackets)

	Rigid^a^	SelfCrit^b^	Narcis^c^	PerfTOT^d^
Rigid^a^				
SelfCrit^b^	.56^f^ (.48^f^)			
Narcis^c^	−.01 (.20)	.20 (.09)		
PerfTOT^d^	.75^f^ (.77^f^)	.89^f^ (.79^f^)	.48^f^ (.57^f^)	
TOA^e^	−.24 (−.42^f^)	−.24 (−.18)	−.25 (−.26^g^)	−.32^g^ (.39^f^)

^a^ Self-critical perfectionism,

^b^ Rigid perfectionism

^c^ Narcistic perfectionism,

^d^Total perfectionism

^e^ Tolerance of ambiguity

^f^ Correlation is significant at the 0.01 level (2-tailed)

^g^ Correlation is significant at the 0.05 level (2-tailed)

Of particular interest was the observed significant decrease in TOA between the pre and in the post-surveys (*p* < 0.01). Perfectionism levels remained stable (*p* > 0.05) for both the total and factor scores (Table 3). The scales showed excellent reliability with Cronbach’s alpha consistently above 0.7 (Table 4).

**Table 3:** Pre and post-clerkship TOA and perfectionism

	Pre Clerkship (n = 51)		Post Clerkship (*n* = 62)						
	Mean	s.d	Mean	s.d.	t-value	Df	*p*-value	95%CI	
SelfCrit^a^	3.22	0.91	3.24	0.86	0.12	111	0.91	−0.31	0.35
Rigid^b^	3.24	0.97	3.12	0.99	0.62	111	0.54	−0.48	0.25
Narcis^c^	1.79	0.59	1.81	0.68	0.15	111	0.88	−0.22	0.26
PerfTOT^d^	2.69	0.59	2.67	0.59	0.13	111	0.90	−0.24	0.21
TOA^e^	59.47	8.26	43.90	7.61	10.41	111	0.00	−18.53	−12.58

*s.d*. Standard deviation, *Df* Degrees of freedom, *CI* Confidence interval

^a^ Rigid perfectionism,

^b^ Self-critical perfectionism,

^c^ Narcistic perfectionism,

^d^Total perfectionism

^e^ Tolerance of ambiguity

**Table 4:** Cronbach’s alpha for each variable used in the assessments

	Pre clerkship	Post Clerkship
SelfCrit^a^	0.829	0.858
Rigid^b^	0.826	0.84
Narcis^c^	0.738	0.796
PerfTOT^d^	0.832	0.837
TOA^e^	0.757	0.701

^a^ Self-critical perfectionism,

^b^Rigid perfectionism

^c^ Narcistic perfectionism,

^d^Total perfectionism

^e^ Tolerance of ambiguity

For the pre survey, there was a significant difference between primary care and “other” group regarding rigid perfectionism (*p* = 0.01) and total perfectionism (*p* = 0.01) (Table 5) with the primary care specialties having lower perfectionism scores. This relationship was not found in the post-survey (*p* > 0.05) (Table 6). There was a non significant relationship between specialties and self-critical perfectionism, narcissistic perfectionism, and TOA (*p* > 0.05). Of note was the decrease in self reported TOA for both groups over the period of the 1 year clerkship.

**Table 5:** Relationship between specialty choices and TOA and perfectionism pre-clerkship

	Primary care (*n* = 28)		Other (*n* = 23)						
	Mean	s.d.	Mean	s.d.	t-value	df	*p*-value	95%CI	
SelfCrit^a^	3.08	0.75	3.40	1.06	1.27	49	0.21	−0.83	0.19
Rigid^b^	2.91	0.98	3.63	0.82	2.80	49	0.01	−1.24	−0.20
Narcis^c^	1.65	0.55	1.96	0.61	1.86	49	0.07	−0.63	0.02
PerfTOT^d^	2.50	0.54	2.92	0.59	−2.62	49	0.01	−0.73	−0.10
TOA^e^	60.04	9.34	58.77	6.86	0.54	49	0.59	−3.44	5.97

*s.d*. Standard deviation, *Df* Degrees of freedom, *CI* Confidence interval

^a^ Self-critical perfectionism,

^b^Rigid perfectionism

^c^ Narcistic perfectionism,

^d^Total perfectionism

^e^ Tolerance of ambiguity

**Table 6:** Relationship between specialty choices and TOA and perfectionism post-clerkship

	Primary care (n = 23)		Other (*n* = 17)						
	Mean	s.d.	Mean	s.d.	t-value	df	*p*-value	95% CI	
SelfCrit^a^	3.05	0.76	3.45	1.01	1.43	38	0.16	−0.97	0.17
Rigid^b^	2.96	0.89	3.38	1.21	1.28	38	0.21	−1.10	0.25
Narcis^c^	1.89	0.71	1.63	0.76	1.13	38	0.27	−0.21	0.74
PerfTOT^d^	2.59	0.56	2.75	0.74	0.76	38	0.45	−0.58	0.26
TOA^e^	44.15	5.38	41.28	9.77	1.19	38	0.24	−2.01	7.76

*s.d*. Standard deviation, *Df* Degrees of freedom, *CI* Confidence interval

^a^ Self-critical perfectionism,

^b^Rigid perfectionism

^c^ Narcistic perfectionism,

^d^Total perfectionism

^e^ Tolerance of ambiguity

## Discussion

The literature is scarce on perfectionism and TOA of medical students and the effect of clinical experience. This study adds to this growing body of literature and hopefully encourages further investigation of how clinical experiences and personality traits impact medical students’ speciality choices and future performance as a physician. Within the limitations of this study, there were some indications that students in this yearlong clinical exposure showed a decrease in their self reported tolerance of ambiguity (TOA) while perfectionism scores remained relatively stable. The perfectionism results were expected given that perfectionism is a relatively stable construct in adults [31]. In contrast, these TOA results were unexpected in relation to previous studies that reported an increase in TOA when participants are exposed to artificially ambiguous scenarios [32]. Cross sectional studies have shown final year medical students have similar TOA compared to those entering clerkship for their first year, and residents reporting higher TOA than medical students [11]. As such, clinical exposure decreasing TOA was an unexpected result. These findings might be indicative of the medical student’s first year of clinical experience where “real” decisions about patients are required in contrast to classroom discussions; however further research is needed to confirm these current results. Previous studies have shown that intolerance of ambiguity (low TOA) has been implicated in anxiety [33]. Should these results hold, a possible area of inquiry might focus on the anxiety cycle.

Anxiety has been described to be maintained through short exposures and avoidance of anxiety inducing situations. Theortically, avoidance allows short term relief, and the short exposures do not allow for applying effective coping skills, which leads to worsening anxiety [34, 35]. Perhaps the decrease in TOA observed at the end of the year long clinical exposure may reflect this anxiety cycle in the medical training context. The frequency of rotation changes may be sensitizing students to ambiguity. At this institution, most rotations last approximately 2 weeks, with the longest lasting 6 weeks. As such, clinical clerks are shifted to different environments more frequently than residents who have rotations lasting months. The frequent changes may not allow medical students to get accustomed to the environment and develop the knowledge and skills to handle varied patient issues, which may leave them in a heightened state of discomfort. It is possible that in other studies, residents may have experienced a longer time span within and between rotations, and as such had more time to become accustomed to the uncertainty and develop coping strategies which would account for increased TOA.

This study demonstrated that those with lower rigid perfectionism were more likely to prefer primary care specialties at the beginning of clerkship. One hypothesis is that rigid perfectionism, which is defined as “our own performance must be perfect”, is appropriately low in those preferring primary care specialties [21]. Primary care specialties are often filled uncertain outcomes, which largely depend on patient compliance to treatment regiments [36, 37]. A physician’s performance in these situations does not have the same impact on patient outcomes and other areas of medical practice. Patient compliance has been shown to be influenced by factors such as a providers’ empathy, in contrast to their medical knowledge [38, 39]. As such, those thriving in primary care must be flexible in their approaches to patient care which is conducive to those with low rigid perfectionism.

### Study strength

The key strength of this study is its repeated sampling of a single cohort at the start and end of their first year of clinical experiences. The two samplings allow a deeper insight into how these factors can change with time and impact the variability experienced in medical training. Previous research has studied differences in ambiguity across levels of medical training through a cross-sectional design which limits their generalizability [11, 12].

### Limitations

While these findings present some interesting preliminary considerations, there are several limitations. While there were two data collection periods with a single cohort, due to the nature of this survey, it was not possible to match participants at the two time points. This limits the extent to which one can infer the effect of clinical exposure on the same persons over time, compared to other confounders which were not controlled for. Another limitation is the variety of clinical exposures. While all students had similar broad specialty exposures, there are variations in the encounters they may have had within a specialty, or in the subspecialty exposures. The anonymous nature of the study hindered capturing such granular data. While individual person level between pre and post assessment could not be matched, there was a substantial overlap between those who took this survey at the start of clerkship and those who completed it at the end of clerkship. With regards to student participation, 50 students responded to the pre-survey and 62 the post-survey. This represents a response rate of 29 and 35% respectively of the larger cohort of students which limits the extent to which the findings can be generalized to this group, even though participation was around 30% which is in keeping with reported response rates of web-based surveys in the medical field [40].

## Conclusions

This study demonstrates a decrease in TOA with clinical experience. This was an unexpected finding and not previously found in the literature. One hypothesis for this finding may be due to a sensitization phenomenon. With regards to specialty choices, at the beginning of clerkship, those who ranked primary care specialties as their most preferred choice reported lower levels of perfectionism, specifically rigid perfectionism. Further research in this field is necessary in order to better understand the impact of these personality traits in the clinical field.

## Data Availability

The datasets used and/or analyzed during the current study are available from the corresponding author on reasonable request.

## Abbreviations

TOA: Tolerance of ambiguity
SelfCrit: Self-critical perfectionism
Narcis: Narcissistic perfectionism
TAMSAD: Tolerance of Ambiguity in Medical Students and Doctors
